# Normal and Abortive Buds Transcriptomic Profiling of *Broccoli ogu* Cytoplasmic Male Sterile Line and Its Maintainer

**DOI:** 10.3390/ijms19092501

**Published:** 2018-08-24

**Authors:** Jinshuai Shu, Lili Zhang, Yumei Liu, Zhansheng Li, Zhiyuan Fang, Limei Yang, Mu Zhuang, Yangyong Zhang, Honghao Lv

**Affiliations:** Institute of Vegetables and Flowers, Chinese Academy of Agricultural Sciences, Key Laboratory of Biology and Genetic Improvement of Horticultural Crops, Ministry of Agriculture, 12 Zhongguancun Nandajie Street, Beijing 100081, China; shujinshuai@caas.cn (J.S.); zhanglilina@126.com (L.Z.); lizhansheng@caas.cn (Z.L.); fangzhiyuan@caas.cn (Z.F.); yanglimei@caas.cn (L.Y.); zhuangmu@caas.cn (M.Z.); zhangyangyong@caas.cn (Y.Z.); lvhonghao@caas.cn (H.L.)

**Keywords:** broccoli, cytoplasmic male sterile, bud abortion, gene expression, transcriptome, RNA-Seq

## Abstract

Bud abortion is the main factor affecting hybrid seeds’ yield during broccoli cross breeding when using *ogura* cytoplasmic male sterile (*ogu* CMS) lines. However, the genes associated with bud abortion are poorly understood. We applied RNA sequencing to analyze the transcriptomes of normal and abortive buds of broccoli maintainer and *ogu* CMS lines. Functional analysis showed that among the 54,753 annotated unigenes obtained, 74 and 21 differentially expressed genes in common were upregulated and downregulated in *ogu* CMS abortive buds compared with *ogu* CMS normal buds, maintainer normal, and abortive buds, respectively. Nineteen of the common differentially expressed genes were enriched by GO terms associated with glycosyl hydrolases, reactive oxygen species scavenging, inhibitor, and protein degradation. Ethylene-responsive transcription factor 115 and transcriptional factor basic helix-loop-helix 137 were significantly upregulated; transcription factors DUO1 and PosF21/RF2a/BZIP34 were downregulated in *ogu* CMS abortive buds compared with the other groups. Genes related to polygalacturonase metabolism, glycosyl hydrolases, oxidation reduction process, phenylalanine metabolism, and phenylpropanoid biosynthesis were significantly changed in *ogu* CMS abortive buds. Our results increase our understanding of bud abortion, provide a valuable resource for further functional characterization of *ogu* CMS during bud abortion, and will aid in future cross breeding of *Brassica* crops.

## 1. Introduction

Bud abortion is a very common biological phenomenon in *Brassica* species. During bud abortion, the buds stop growing, convert from green into yellow progressively from the base to the top of the bud, and eventually wither off before flowering [1,2], which is detrimental for hybrid seeds yield during cross breeding. In recent years, bud abortion has attracted considerable attention in *Brassica* species cross breeding [3,4,5], as it reduces the hybrid seed yield production of many *Brassica* crops and also affects the efficiency of *Brassica* crops cross breeding. In the past decade, to solve the bud abortion problem, many studies have investigated the factors that cause bud abortion [1,2,3,4,5,6,7,8]. However, most of the studies focused on morphology and anatomy, and few studies have reported the factors contributing to *Brassica* bud abortion at the molecular level. Therefore, the fundamental mechanism of bud abortion is poorly understood.

High-throughput sequencing technologies have played important roles in revealing the molecular mechanisms of various organismal biological processes. RNA sequencing (RNA-Seq) technology is an important high throughput sequencing technology that produces functional genomic data for non-model plants that lack genomic sequence data. RNA-Seq technology has been widely applied to assist in determining differentially expressed genes (DEGs) involved in different biological processes in many species and may be a promising method to address the genes associated with bud abortion in *Brassica* species. Surprisingly, no studies on bud abortion using RNA-Seq technology have been documented so far.

*Brassica* species display obvious heterosis, and using cytoplasmic male sterile (CMS) lines to produce hybrid seeds is an important approach that uses this heterosis. Broccoli (*Brassica oleracea* var. *italica*) is an important vegetable crop and plays a vital role in the vegetable production industry worldwide. More importantly, broccoli is also used to produce health products and develop anti-cancer drugs [9,10,11]. Thus, broccoli is the most important economical *Brassica* vegetable crop. However, a large problem exists in broccoli cross breeding: the hybrid seed purity cannot reach 100% when using inbred lines or self-incompatible lines to produce hybrid seeds. To improve the purity of broccoli hybrid seeds, many breeders execute wide crosses between broccoli cultivars and other *Brassica* CMS materials to transfer CMS genes. Our group used multiple broccoli inbred lines to with the cabbage CMS material *ogura* CMSR_3_629 (*ogu* CMS), which was introduced by the Asgrow Seed Co. (USA), which has been used to breed a plurality of broccoli CMS lines with a 100% sterile rate. However, the obtained CMS lines often show serious bud abortion, which leads to delayed flowering time and sharply reduced amounts of flowers, some serious bud abortion CMS lines cannot flower [5]. These problems limit the use of the CMS lines considerably and have reduced their breeding efficiency dramatically. So far, several studies have focused on the causes of bud abortion, e.g., the *RsVPE1* gene encodes a vacuolar processing enzyme that is involved in radish floral bud abortion under heat stress [1]. The expression of stress response, energy metabolism, amino acid synthesizing and processing, signal transduction, disease resistance and senescence, transcription and translation, and transmembrane transport-related genes were different between normal and abortive buds, as detected by cDNA-amplified length polymorphism technology [6,7,8]. However, as far as we know, no study has investigated the molecular events occurring in bud abortion based on high throughput sequencing during broccoli cross breeding, or any other crop, thus little information is available concerning the genes involved in bud abortion. Thus, it is necessary to determine the elements involved in broccoli bud abortion at the molecular level, which will increase our understanding of the molecular events involved in bud abortion of broccoli and other crops. The objectives of this study were to identify the functional genes involved in bud abortion and to determine the genes expression characteristics related to broccoli bud abortion, based on RNA-Seq technology.

## 2. Results

### 2.1. High-Throughput Transcriptome Sequencing and Unigene Assembly

**Figure 1:**
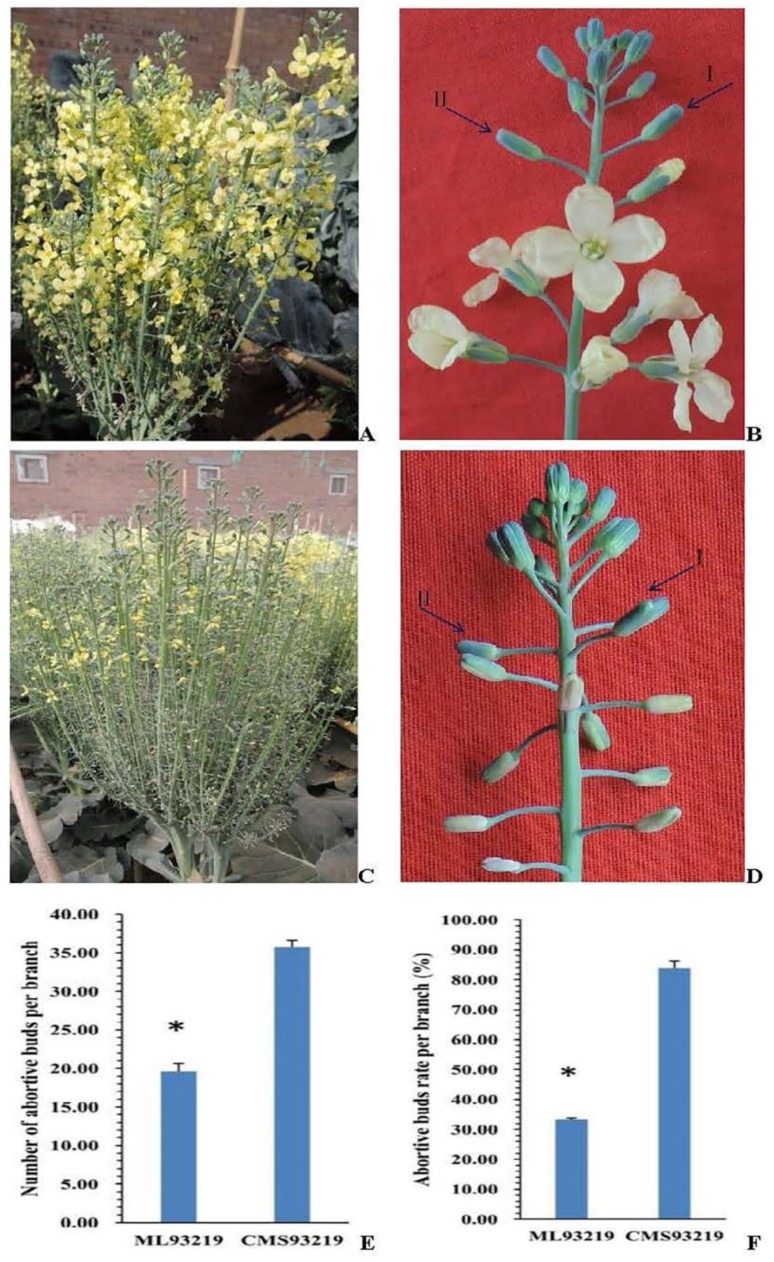
Morphological characterization of two broccoli lines with different degrees of bud abortion. (**A**,**B**) ML93219, (**C**,**D**) CMS93219. B-I and B-II represent the normal and abortive bud of ML93219, respectively. D-I and D-II represent the normal and abortive bud of CMS93219, respectively. The bars in (**E**,**F**) represent the standard deviation (*n* = 15). Asterisks indicate that the average number of abortive buds and the abortive buds rate per branch are very significantly different between ML93219 and CMS93219 (unpaired *t* test, *p* < 0.01).

To explore gene expression and gene networks that control bud abortion of *ogu* CMS lines in broccoli, we performed RNA-Seq analysis of normal and abortive buds from *ogu* CMS line CMS93219 and its maintainer line ML93219. The cytoplasmic male sterility original of CMS93219 was cabbage CMS material *ogu* CMSR_3_629 and the backcross paternal was broccoli ML93219. CMS93219 was stabilized via sixteen generations of backcrossing before this study. There were no visible differences between CMS93219 and ML93219 in terms of the plants and the heading morphologies of the harvested material, except that CMS93219 showed significant bud abortion and ML93219 showed only slight bud abortion before or at the early anthesis stage (Figure 1A–D). The average number of abortive buds per branch of CMS93219 was 35.73 throughout the whole growth phase, accounting for 83.88% of the total bud number, which is extremely significantly higher than the 19.60 per branch (33.33%) in ML93219 (*t*-test, *p* < 0.01) (Figure 1E,F). Neither the abortive nor the normal buds of CMS93219 contained pollen, while the aborted buds of ML93219 contained less pollen than normal buds. Therefore, bud abortion and pollen abortion character were closely connected between ML93219 and CMS93219, and pollen abortion may promote bud abortion. Gene expression changes are usually associated with morphologic changes; therefore, we chose the maximum buds stage (around 3 days before anthesis) for transcriptome analyses (Figure 1B-I,B-II,D-I,D-II).

Twelve cDNA libraries from normal buds and abortive buds of the ML93219 and CMS93219, containing three biological replicates for each sample, were subjected to Illumina sequencing. After filtering invalid reads and data cleaning, 663,701,918 clean reads containing 99,555,287,700 nucleotides (99.6 Gb), with a mean length of 150 bp, were obtained. The Q20 and GC percentage were 92.93–94.33% and 46.46–47.12%, respectively (Table 1).

After assembling the clean reads, 97,347 transcripts (91,137,323 nucleotides) and 66,050 unigenes (51,896,834 nucleotides) were obtained. The average length of the transcripts and unigenes were 936 and 786 bp, respectively (Table 2).

### 2.2. Gene Annotation and Functional Classification

A total of 54,753 unigenes (82.90%) were annotated in at least one database and 6070 unigenes (9.19%) were annotated in seven databases. In the NCBI non-redundant protein sequences (Nr), NCBI non-redundant nucleotide sequences (Nt), KEGG Ortholog (KO), a manually annotated and reviewed protein sequence database (SwissProt), protein family (Pfam), Gene Ontology (GO), eukaryotic ortholog groups (KOG) databases, 44,294 (67.06%), 50,157 (75.93%), 12,403 (18.77%), 29,861 (45.2%), 24,660 (37.33%), 30,285 (45.85%) and 12,492 (18.91%) unigenes were annotated, respectively (Table 3).

**Figure 2:**
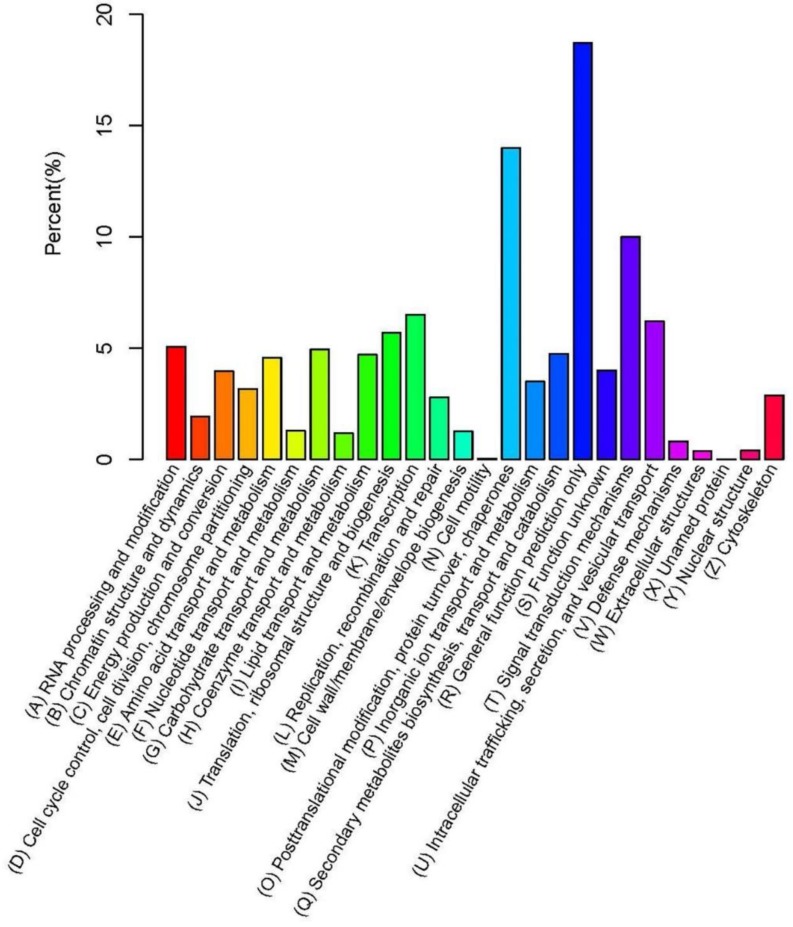
Clusters of eukaryotic orthologous groups (KOG) functional classification of the broccoli bud transcriptome.

We used the KOG functional annotation system for the assembled unigenes to obtain clues to the functions of the unigenes. As a result, 12,492 unigenes have defined, specific protein functions, accounting for 22.82% of the total annotated unigenes and involving 26 KOG functional classes. The five largest categories were “General function prediction only”, “Posttranslational modification, protein turnover, chaperones”, “Signal transduction mechanisms”, “Transcription”, “Intracellular trafficking, secretion, and vesicular transport”. Two categories, “Unnamed protein” and “Cell motility”, accounted for the lowest percentages (Figure 2).

**Figure 3:**
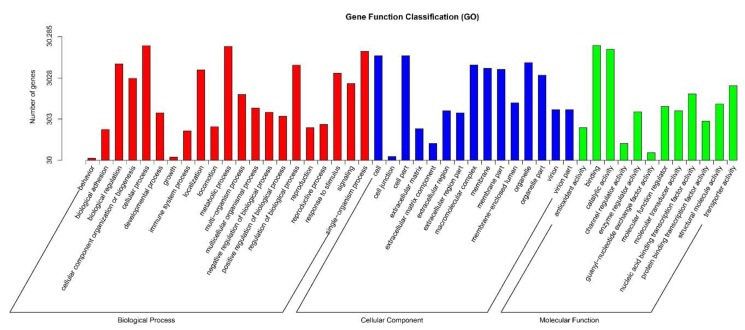
Gene Ontology (GO) classifications of the broccoli bud transcriptome.

To classify the functions of the unigenes, GO assignments were used based on the annotations from the Nr and Pfam databases. The results showed that 30,285 unigenes could be categorized into 57 functional groups, which were separated into three main categories: biological process (25 subcategories), cellular component (18 subcategories), and molecular function (14 subcategories) (Figure 3). The “cellular process,” “metabolic process,” and “single-organism process” were the major subcategories of the biological processes category; “cell” and “cell part,” “binding,” and “catalytic activity” were remarkable in the cellular component and molecular function categories, respectively. The classification result indicated that biological processes play a notable role during broccoli bud development, while the terms “biological phase,” “cell aggregation,” “synapse,” “synapse part,” “receptor regulator activity,” and “metallochaperone activity” were rare.

To predict the activated biochemical pathways in broccoli buds, the unigenes were annotated against the KEGG database (*e*-value = 1 × 10^−10^). A total of 264 KEGG pathways represented by 12,403 unigenes were obtained (Table S1). The main pathways were “Biosynthesis of amino acids [ko01230] (454 unigenes),” “Plant hormone signal transduction [ko04075]” (447 unigenes), “Carbon metabolism [ko01200]” (433 unigenes), and “Starch and sucrose metabolism [ko00500]” (366 unigenes). Moreover, the pathways such as “Endocytosis [ko04144],” “RNA degradation [ko03018],” “ubiquitin mediated proteolysis [ko04120],” “Fatty acid degradation [ko00071],” “Valine, leucine and isoleucine degradation [ko00280],” “Proteasome [ko03050],” “Lysine degradation [ko00310],” and “Apoptosis [ko04210],” which contained 235, 187, 184, 82, 79, 77, 49, and 31 unigenes, respectively, which indicated that protein degradation and cell death were associated with bud abortion.

### 2.3. Genes Express Differences and DEGs Clustering

**Figure 4:**
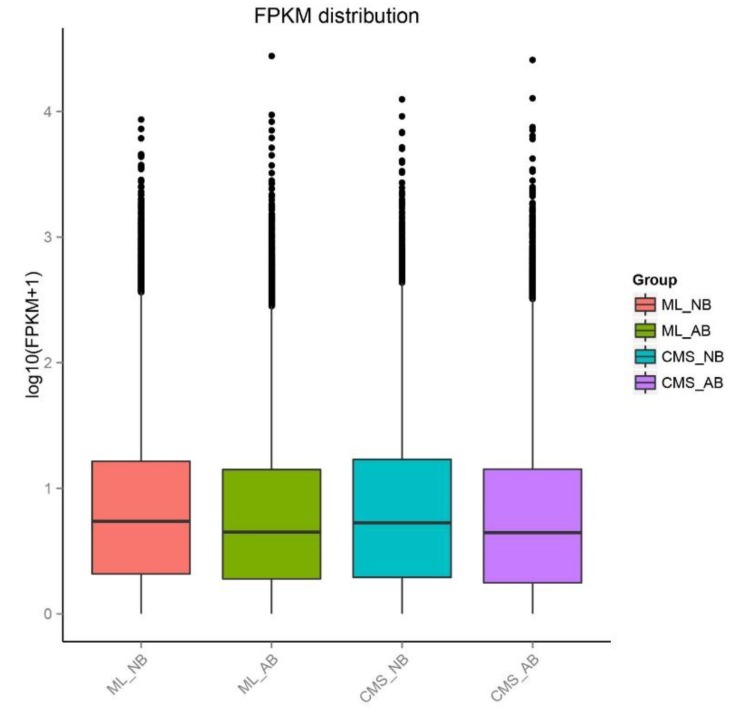
Boxplot of Fragments per kb per million fragments (FPKM) distribution for the four samples. Five statistics are represented by different regions of the Boxplot; from the top down they are the maximum, upper quartile, median, lower quartile, and minimum, respectively.

**Figure 5:**
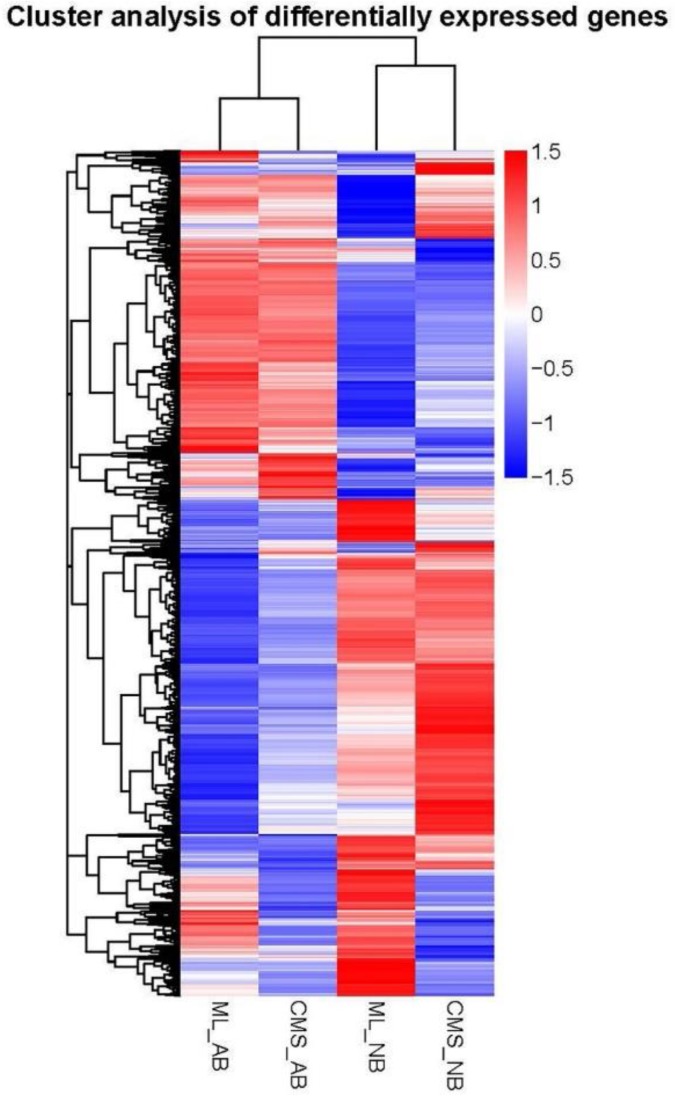
Cluster analysis of differentially expressed genes among the four samples. Heatmap of differentially expressed genes among the four samples. Red indicates high expression, and blue indicates low expression. Color from red to blue represents descending log10 (FPKM + 1).

The 97,347 assembly unigenes sequences obtained from de novo assemble of a merged set of 663,701,918 clean reads were used as the reference. We acquired the number of readcounts that aligned to each unigene and calculated the expected number of Fragments Per Kilobase of transcript sequence per Millions base pairs sequenced (FPKM) value to estimate the expression levels of the unigenes. The FPKM distribution and heatmap indicated the presence of many DEGs among the four samples, and the expression patterns of aborted buds of ML93219 (ML_AB) and aborted buds of *ogu* CMS93219 (CMS_AB), and normal buds of ML93219 (ML_NB) and normal buds of *ogu* CMS93219 (CMS_NB) were more similar, respectively (Figure 4 and Figure 5). Changes in the DEGs indicated that the abundances of transcripts were different at the same stage of normal and abortive buds development between ML93219 and CMS93219.

### 2.4. DEGs in Normal and Abortive Buds of ogu CMS

**Figure 6:**
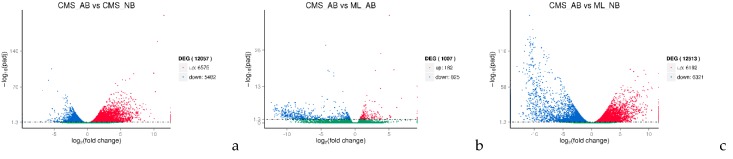
Differentially expressed genes (DEGs) between normal and abortive buds from ML93219 and CMS93219. (**a**) DEGs between CMS AB and CMS NB. (**b**) DEGs between CMS AB and ML AB. (**c**) DEGs between CMS AB and ML NB. The *x*-axis indicates the log_2_ (fold change) between the two samples. The *y*-axis indicates −log_10_ (padj) (*p*-adjusted). The scatter points in the figure represent individual genes, the green dots indicate genes with no significant differences, the red dots indicate up-regulated genes with significant differences, and the blue dots indicate down-regulated genes with significant differences. The screening condition for DEGs is padj < 0.05.

To explore the reference sample CMS_AB gene expression levels, the gene expression variations were determined using three comparisons, between CMS_AB and CMS_NB, between CMS_AB and ML_NB and between CMS_AB and ML_AB. Compared with CMS_NB, there were 6575 and 5482 genes up- and downregulated in CMS_AB, respectively. Compared with ML_NB, in CMS_AB, there were 6192 and 6321 genes that were up- and downregulated, respectively. Compared with ML_AB, there were 182 and 825 genes that were up- and downregulated in CMS_AB, respectively (Figure 6a–c).

**Figure 7:**
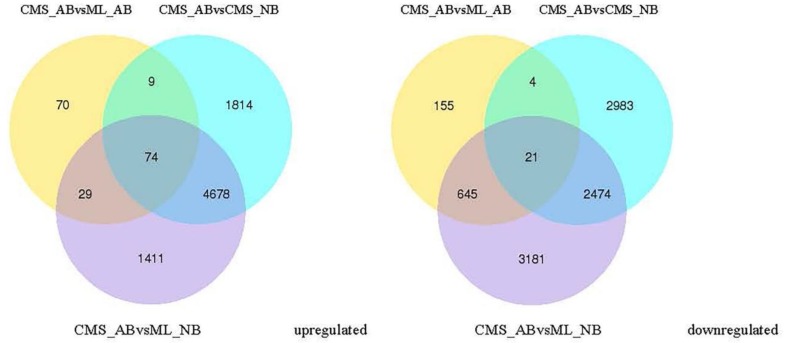
DEGs between normal and abortive buds from broccoli maintainer and *ogu* cytoplasmic male sterile (CMS) lines.

To determine the key genes involved in *ogu* CMS line bud abortion process, we compared the DEGs of normal and abortive buds from ML93219 and CMS93219 to define the differentially expressed genes in common. In CMS_AB, 74 and 21 genes were upregulated and downregulated, respectively, compared with CMS_NB, ML_NB and ML_AB (Figure 7; Tables S2–S5), which are most likely associated with *ogu* CMS bud abortion in broccoli.

### 2.5. Validation of RNA-Seq Data by Quantitative Real-Time Reverse Transcription PCR (qRT-PCR)

**Figure 8:**
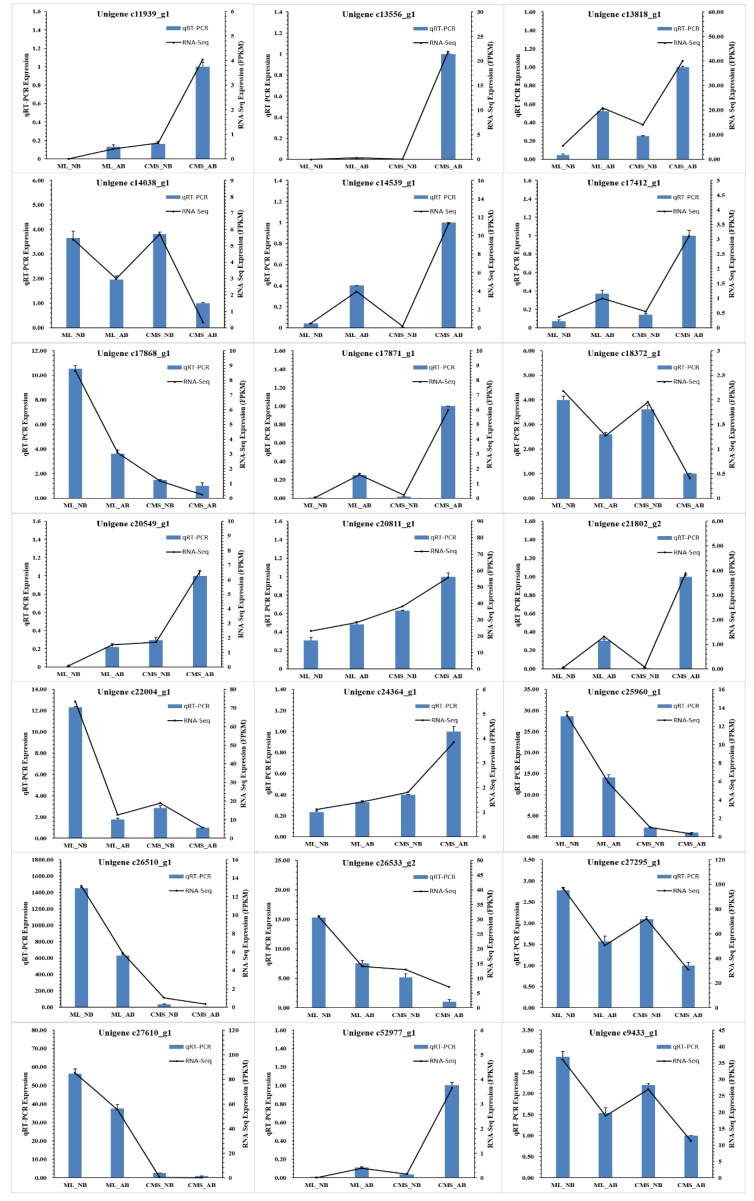
Verification of the DEGs by qRT-PCR. Eleven DEGs with higher expression and ten DEGs with lower expression in CMS_AB were selected for qRT-PCR validation. The relative expression level of each gene was expressed as the FPKM among four samples in the RNA-Seq data (black line) and qRT-PCR data (blue bar). To normalize the expression data, the broccoli *β-actin* gene was used as the internal control. The bars represent the standard deviation.

We performed qRT-PCR to verify the DEGs identified by RNA-Seq, using the same samples that were used for the RNA-Seq analysis. Among the 21 randomly selected DEGs based on the expression level fold-change and differences in expression, 11 genes displayed higher expression quantification and 10 genes showed lower expression quantification in CMS_AB, compared with CMS_NB, ML_NB, and ML_AB. All 21 genes showed the same expression patterns in qRT-PCR as they did in the RNA-Seq databases experiment, indicating the high reliability of our RNA-Seq data (Figure 8).

### 2.6. Ogu CMS Bud Abortion-Related Genes in Broccoli

To further understand the function and biological process of the DEGs, GO term (corrected *p* < 0.05) and KEGG pathway (corrected *p* < 0.05) enrichment were performed to analyse the 95 differentially coexpressed genes. Nineteen were enriched and the most significantly enriched GO terms were “cell wall organization” and “external encapsulating structure organization” (corrected *p* = 3.26 × 10^−5^) in the biological process (BP) group, “extracellular region” (corrected *p* = 0.003937) in the cellular component (CC) group and “polygalacturonase activity” (corrected *p* = 0.015471) in the molecular function (MF) group (Table 4). The GO terms of “cell wall organization or biogenesis” and “extracellular region” contained 10 and 13 genes, respectively, and were the biggest categories in the biological process and cellular component groups (Table 4), respectively. Only “polygalacturonase activity” GO terms in molecular function group was enriched and contained 4 genes (Table 4). Furthermore, there were 8 same genes were enriched in “sucrose metabolic process,” “starch metabolic process,” “disaccharide metabolic process,” “cellular glucan metabolic process,” “glucan metabolic process” and “oligosaccharide metabolic process” (Table 4); 5 same genes were enriched between “external encapsulating structure” and “cell wall” (Table 4). “Phenylalanine metabolism” and “Phenylpropanoid biosynthesis” (K00430) contained the same three genes (Gene ID: c20440_g1, c20661_g1, c9433_g1) that were significantly enriched in KEGG pathways; meanwhile, the three genes were enriched by GO enrichment (Table 4 and Table 5).

**Figure 9:**
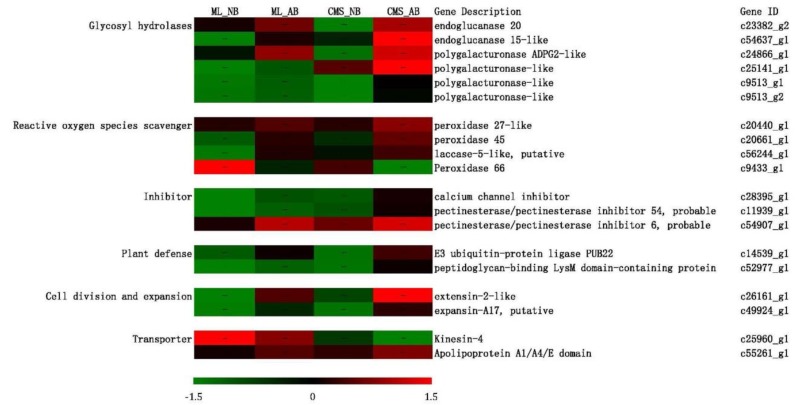
Heatmap of the 19 enriched common differentially expressed genes in normal and abortive broccoli buds. The bar represents the expression levels for each gene (log_10_ (FPKM + 1)) in the ML_NB, ML_AB, CMS_NB, and CMS_AB groups, as indicated by red or green rectangles. Red means upregulation of genes and green means downregulation.

Based on the enrichment results and functional annotation of the 19 enriched differential expressed genes in common by GO term, we found that “Glycosyl hydrolases” and “Reactive oxygen species (ROS) scavenger” related genes accounted for a high proportion (Figure 9). In addition, “Inhibitor,” “Plant defense,” and “Cell division and expansion” related genes were also significantly upregulated in CMS_AB and “Transporter” related gene Kinesin-4 downregulated in CMS_AB (Figure 9). Furthermore, five of the differentially expressed genes in common (GI: c11939_g1, c14539_g1, c25960_g1, c52977_g1, c9433_g1) enriched by GO terms were verified by qRT-PCR, and had significantly different expression between the abortive buds and normal buds of CMS93219 (Figure 8). These results suggested that most of the differentially expressed genes in common found in our study are required for *ogu* CMS bud abortion in broccoli.

### 2.7. Transcription Factors Are Involved in Broccoli ogu CMS Bud Abortion Control

Among the 95 common differentially expressed genes, four transcription factors were identified, including predicted transcription factor basic helix-loop-helix (bHLH) 137 (GI: c13818_g1), ethylene-responsive transcription factor (ERF) 115 (GI: c21802_g2), transcription factor DUO1 (GI: c18372_g1), and PosF21/RF2a/BZIP34 (GI: c14038_g1), which were distributed in four different gene families. Compared with CMS_NB, ML_NB, and ML_AB, transcription factors bHLH137 ERF115 were upregulated in CMS_AB, suggesting that these transcription factors may function as positive regulators in *ogu* CMS bud abortion in broccoli. Conversely, transcription factors DUO1 and PosF21/RF2a/BZIP34 were downregulated in CMS_AB, suggesting that they are negative regulators. Moreover, the expression quantifications of the four transcription factors were confirmed by qRT-PCR (Figure 8), suggesting that the four transcription factors play key roles in *ogu* CMS bud abortion in broccoli.

## 3. Discussion

The bud abortion phenomenon is a very complex bioprocess and demands many molecular events during bud development. In this study, we used RNA-Seq technology to explore the genes involved in *ogu* CMS bud abortion process and to provide a comprehensive analysis of the genes involved in *ogu* CMS bud abortion control in broccoli. Compared with CMS_NB, ML_NB, ML_AB, there were 6575, 182, and 6192 genes that were upregulated and 5482, 825 and 6321 genes that were downregulated in CMS_AB, respectively (Figure 6a–c), among which 74 genes were significantly upregulated and 21 genes were significantly downregulated equally in CMS_AB with serious bud abortion (Figure 7; Tables S2–S5). qRT-PCR proved that our RNA-Seq data was highly reliable (Figure 8). Functional categories of the common differentially expressed genes by GO term enrichment analysis showed that gene associated with cell wall composition and metabolism, such as cell wall organization, cell wall organization or biogenesis; extracellular region, such as external encapsulating structure organization; sugar metabolism, such as sucrose metabolic process, starch metabolic process, disaccharide metabolic process, cellular glucan metabolic process, glucan metabolic process, oligosaccharide metabolic process related genes were strongly induced in the buds abortion of broccoli (Table 4). These results indicated that bud abortion may be closely related to cell wall organization, external encapsulating structures and sugar metabolism related genes play an important role in bud abortion in broccoli. Our results were consistent with the differential expression genes obtained by cDNA-AFLP technique in radish and cabbage between normal bud and dead bud [6,7,8].

### 3.1. Genes Related to Programmed Cell Death Are Involved in Bud Abortion

Programmed cell death (PCD) is an important physiological process in single cells and penetrates the whole plant life cycle, which can help plants to control and organize the destruction of non-functional or redundant damaged cells [13,14,15]. Although PCD is a natural result of ageing, it may be switched on by environmental stress or irregular development in plants [15]. Increases in caspase-like proteases [16] and ROS [17,18] activities of metacaspase gene family related genes [19] are the important features of PCD. In this study, several genes associated with PCD were significantly changed in *ogu* CMS abortive buds compared with normal buds and the maintainer abortive buds (CMS_NB, ML_NB and ML_AB). Among the 8833 differential expression genes between ML_AB vs. ML_NB and CMS_AB vs. CMS_NB, there was 11.75% participate in the redox process, 11.08% with redox enzyme activity and 55.38% with catalytic activity, indicating that redox process involved in bud abortion in broccoli. ROS scavengers related genes, such as: peroxidase 27-like and peroxidase 45, l-lactate dehydrogenase, laccase-5-like and fatty acyl-CoA reductase 7 (Table S5), which were all upregulated in abortive buds compared with normal buds, suggesting that higher levels of ROS were produced in abortive buds and ROS scavenger-related genes were closely related with broccoli bud abortion. Moreover, 13 caspase-like and metacaspase activity genes involved in cell apoptosis were also discovered, such as metacaspase-1, metacaspase-3, metacaspase-5, metacaspase-6, metacaspase-7, metacaspase-9, pentapeptide repeats protein, caspase recruitment domain, BTB/POZ domain-containing protein POB1. The expression of these genes in abortion buds was significantly higher than that of normal buds suggesting that bud abortion was related with PCD.

### 3.2. Glycosyl Hydrolases, Inhibitors and Plant Defence Related Genes Are Implicated in Bud Abortion

In this study, several glycosyl hydrolase-related genes were determined as significantly upregulated in *ogu* CMS abortive buds, such as endoglucanase 20, endoglucanase 15-like, polygalacturonase ADPG2-like and polygalacturonase-like. However, polygalacturonase plays an important role during the life cycle of cell separation, being involved in cell wall modification, abscission and dehiscence in *Arabidopsis thaliana* [20,21], and endoglucanase is involved in cell wall biogenesis or degradation, cellulose degradation and polysaccharide degradation [22], which suggested that glycosyl hydrolase-related genes were required for *ogu* CMS bud abortion in broccoli. Pectinesterase/pectinesterase inhibitors modify cell walls via demethylesterification of cell wall pectin, which negatively regulates catalytic activity. As a voltage-gated inward-rectifying Ca^2+^ channel (VDCC) across the vacuole membrane, the calcium channel is an essential components of the slow vacuolar (SV) channel and is the major ROS-responsive Ca^2+^ channel, which is the possible target of Al-dependent inhibition and is involved in the regulation of stomatal movement [23,24]. In our study, pectinesterase/pectinesterase inhibitor 6 and 54 probable and calcium channel inhibitor-related genes were highly expressed in *ogu* CMS abortive buds, indicating that these genes may be important in controlling *ogu* CMS bud abortion. Moreover, E3 ubiquitin-protein ligase and peptidoglycan-binding LysM domain-containing protein both play important roles in the plant defence response [23,25,26,27,28,29,30] and nicotianamine synthase is involved in the cellular response to ethylene stimulus [31]: these genes were distinguished significantly upregulated in *ogu* CMS abortive buds, suggesting their likely involvement in *ogu* CMS bud abortion.

### 3.3. Transcription Factors Associated with Bud Development

Transcription factors are critical to regulate gene expression during plant development and in response to biotic and abiotic stresses [32,33,34,35,36,37,38]. In the present study, predicted transcription factors bHLH137, ERF115, DUO1 and PosF21/RF2a/BZIP34 were obviously changed in abortive broccoli buds compared with normal buds. Interestingly, the genes encoding stress-responsive transcription factors ERF115 and gibberellin-responsive transcription factors bHLH137 showed similar expression patterns: both were significantly upregulated in abortive buds (ML_AB and CMS_AB) compared with normal buds (ML_NB and CMS_NB), and in *ogu* CMS abortive buds (CMS_AB), they were significantly upregulated compared with maintainer abortive buds (ML_AB) (Figure 8). Conversely, the genes encoding transcription factors DUO1 and PosF21/RF2a/BZIP34, which are positive regulators of transcription showed similar expression patterns: both were significantly downregulated in abortive buds, and in *ogu* CMS abortive buds they were significantly downregulated compared with maintainer abortive buds (Figure 8). ERF 115 as a transcriptional activator of the phytosulfokine PSK5 peptide hormone family that binds to the GCC-box pathogenesis-related promoter element and limit quiescent center cell division activity when surrounding stem cells are damaged and is also a proteolytic target of the APC/C-FZR1 complex [39]. bHLH transcription factors belong to a family of transcriptional regulators and have a range of different roles in plant cells and tissue development, as well as in plant metabolism [40]. Transcription factor DUO1 could be involved in pollen sperm cell differentiation [41]. PosF21/RF2a/BZIP34 is a transcription factor with an activatory role [42,43], which might be involved in the sporophytic control of cell wall patterning and gametophytic control of pollen development, and play a role in the control of metabolic pathways regulating cellular transport and lipid metabolism [44,45]. Therefore, transcription factors play a key role in the complex regulatory networks of *ogu* CMS bud abortion.

### 3.4. Molecular Mechanisms Associated with CMS

Previous studies have shown that the genes involved in reactive oxygen species (ROS) homeostasis or antioxidative system balance may be important factors contributing to pollen abortion in cotton [46] and wheat [47]. Carbohydrate and energy metabolisms, oxidation-reduction system and phenylpropanoid metabolism pathways related genes may be important factor for CMS in soybean [48], rapeseed [49], cabbage [50], onion [51] and wheat [52]. Male sterility might be related to energy metabolism turbulence, excessive ethylene synthesis, and suffocation of starch synthesis in pepper [53]. In addition, pentatricopeptide repeat proteins, heat shock proteins, stress proteins, MYB, bHLH and heat shock transcription factors and anther development related genes may be the candidates for pollen abortion in *Brassica* crops [49,50,54]. In this study, we found that genes related to polygalacturonase metabolism, glycosyl hydrolases, oxidation reduction process, phenylalanine metabolism, phenylpropanoid biosynthesis were significantly changed in *ogu* CMS abortive buds compared with the other groups. Ethylene-responsive transcription factor 115 and transcriptional factor basic helix-loop-helix 137 were both significantly upregulated in *ogu* CMS abortive buds. Therefore, our results were basically consistent with the results of previous studies [46,47,48,49,50,51,52,53,54], and the genes discovered related to energy metabolism, oxidation reduction process and phenylpropanoid biosynthesis, ethylene-responsive transcription factor 115 and transcriptional factor basic helix-loop-helix 137 may be important factors contributing to *Broccoli ogu* CMS pollen abortion and bud abortion. Further experiments are needed to elucidate the molecular mechanisms of these genes that lead to broccoli CMS and bud abortion.

## 4. Materials and Methods

### 4.1. Plant Materials

Broccoli (*Brassica oleracea* var. *italica*) maintainer ML93219 (showing slight bud abortion) and *ogu* CMS93219 (serious levels of bud abortion) were bred by the Institute of Vegetables and Flowers, Chinese Academy of Agricultural Sciences. The backcross paternal line of *ogu* CMS93219 was ML93219 and the number of backcross generations was sixteen. In the spring of 2015, the plants were grown in an experimental greenhouse at the Institute of Vegetable and Flowers, Chinese Academy of Agricultural Sciences, Changping (Beijing, China). We handled the main bouquet using the approach proposed by Shu et al. [55]. When the plants began to flower, four kinds of bud samples, ML_NB, ML_AB, CMS_NB and CMS_AB, were collected and labelled with three biological replicates. To ensure the integrity of the sample RNA, isolated buds were immediately frozen in liquid nitrogen and stored at −80 °C before RNA extraction.

### 4.2. RNA Extraction and Quality Testing

Total RNA was extracted using an EASYspin Plus Plant RNA-38 Kit, according to the manufacturer’s instructions (Juhuatech Co., Ltd., Beijing, China). The integrity and purity of the RNA samples were determined by 1% agarose gels electrophoresis, and the RNA concentration was measured by Qubit^®^ RNA Assay Kit in Qubit^®^ 2.0 Flurometer (Life Technologies Corporation, Carlsbad, CA, USA) and the integrity of the RNA was assessed by an RNA Nano 6000 Assay Kit of the Agilent Bioanalyzer 2100 system (Agilent Technologies Inc., Santa Clara, CA, USA).

### 4.3. RNA-Seq Library Construction and Illumina Sequencing

Twelve strand-specific RNA-Seq libraries were constructed with cDNA fragments of 250–300 bp in length. An Illumina TruSeq PE Cluster Kit v3-cBot-HS on a cBot Cluster Generation System was then used to cluster the samples, according to the manufacturer’s instructions. After cluster generation, the libraries were sequenced on an Illumina Hiseq™ 4000 system and reads were generated.

### 4.4. RNA-Seq Data Quality Control and Transcriptome de Novo Assembly

We obtained clean reads by removing the adaptor reads, unknown sequences “N” (reads containing more than 10% unknown nucleotides), low quality reads (reads containing more than 50% bases with *Q*-value ≤ 5) from the raw data. The Q20, Q30 and GC-content were then calculated based on the clean reads. The high quality clean reads were used for downstream analyses. Transcriptome *de novo* assembly was executed using Trinity [56] with min_kmer_cov set to 2 by default and other parameters set at their defaults. After assembly, the longest transcripts of each gene were selected as the unigenes.

### 4.5. Unigene Function Annotation

We annotated the unigenes based on seven databases, NCBI blast (2.2.28+) was used to search against the Nr (*E*-value = 1 × 10^−5^), Nt (*E*-value = 1 × 10^−5^), Swiss-Prot (*E*-value = 1 × 10^−5^) and KOG databases (*E*-value = 1 × 10^−3^). The unigenes were divided into 26 groups and their participation in different metabolic pathways based on KOG annotation was assessed. Pfam annotated was determined using the HMMER 3.0 package [57], hmmscan (*e*-value = 0.01). GO annotations for the unigenes were determined by Blast2GO v2.5 [12] with the self-write script (*e*-value = 1 × 10^−6^) based on the annotation result of Nr and Pfam, which has three ontologies: molecular function, cellular component and biological process [58]. KEGG [59] related annotations were identified by the KAAS and KEGG Automatic Annotation Server [60] (*E*-value = 1 × 10^−10^) to determine the metabolic pathway of unigenes.

### 4.6. Analysis of DEGs

Alignment results of bowtie were counted by RSEM [61]. FPKM [62] values were used to calculate the gene expression levels of the four groups of normal and abortive buds from the maintainer and *ogu* CMS lines. FPKM has become the most commonly used method to estimate the level of gene expression and takes into account the effects of sequencing depth and gene length on the calculation of gene expression [62]. There were three biological replicates; therefore, the calculated gene expression could be used directly to compare the gene expression between samples. Referring to the statistical method of Storey and Tibshirani [63], |log_2_Fold change| > 1 and *p*-adjusted < 0.05 were set as the threshold for significantly differential expression. *p*-Values were adjusted to control the false discovery rate, referring to Benjamini and Hochberg’s approach [64]. Then, based on the Wallenius non-central hyper-geometric distribution [65], GO and KEGG functional enrichment analysis of the DEGs was executed by the GOseq [66] and KOBAS software [60], respectively.

### 4.7. qRT-PCR Validation

qRT-PCR analyses with the three biological replicates samples used for RNA-Seq were performed to verify the DGEs results. Twenty-one common differentially expressed genes were randomly selected that accounted for about 22.1% of the 95 common differentially expressed genes. Specific primers were designed using the Primer-BLAST tool (available online: http://www.ncbi.nlm.nih.gov/tools/primer-blast/index.cgi? LINK _LOC=BlastHome) in NCBI and synthesized by Sangon Biotech Co., Ltd. (Shanghai, China). cDNAs were reverse transcribed from total RNA using a PrimeScript RT reagent Kit (Takara, Dalian, China). qRT-PCR was carried out according to the SYBR PrimeScript RT-PCR Kit manufacturer specifications (Takara) on an ABI Prism^®^7900 Real-Time PCR System (Applied Biosystems, Foster City, CA, USA). To normalize the gene expression data, we used the broccoli *β-actin* gene as an internal standard [67]. The 2^−ΔΔ*C*t^ method [68] was used to determine the relative expression of genes. The standard deviation was calculated based on the three biological replicates. The specific primers sequences are listed in Table S6.

## 5. Conclusions

In this study, we found that buds abortion was related with polygalacturonase metabolism, glycosyl hydrolases, oxidation reduction process, phenylalanine metabolism, and phenylpropanoid biosynthesis. Moreover, 19 common differentially expressed genes associated glycosyl hydrolases, reactive oxygen species scavenging, inhibitor, plant defense, cell division and expansion, transporter, and four transcriptional factors—ethylene-responsive transcription factor 115, transcriptional factor basic helix-loop-helix 137, transcription factors DUO1, and PosF21/RF2a/BZIP34—may be associated with *ogu* CMS abortive buds. In conclusion, our results not only increased our understanding of *ogu* CMS bud abortive mechanisms and provided a valuable resource for further functional characterization of *ogu* CMS bud abortion, but also laid the foundation for molecular breeding to overcoming bud abortion in broccoli, as well as other *Brassica* crops in the future.

## Figures and Tables

**Table 1:** Summary of broccoli bud transcriptome sequencing data.

Sample Name	Raw Reads	Clean Reads (Clean/Raw)	Clean Bases (Gb)	Q20 (%)	GC Content (%)	Mapped Reads (Mapped/Clean)
ML_NB1	57,767,246	55,194,896 (95.55%)	8.28	94.30	46.93	41,252,694 (74.74%)
ML_NB2	44,577,570	43,849,450 (98.37%)	6.58	92.93	46.46	32,614,176 (74.38%)
ML_NB3	61,251,852	59,738,162(97.53%)	8.96	94.03	46.96	44,511,290 (74.51%)
ML_AB1	61,588,740	60,493,582 (98.22%)	9.08	94.32	46.73	45,965,016 (75.98%)
ML_AB2	54,823,836	53,530,342 (97.64%)	8.02	93.90	46.74	40,115,268 (74.94%)
ML_AB3	58,528,852	56,475,844 (96.49%)	8.48	94.33	46.81	42,816,816 (75.81%)
CMS_NB1	54,455,258	53,170,054 (97.64%)	7.98	94.23	47.02	39,776,946 (74.81%)
CMS_NB2	53,265,514	52,284,140 (98.16%)	7.84	93.97	47.12	38,965,364 (74.53%)
CMS_NB3	51,816,268	50,884,086 (98.20%)	7.64	93.79	46.91	37,800,172 (74.29%)
CMS_AB1	64,340,176	63,146,694 (98.15%)	9.48	94.16	47.04	47,846,088 (75.77%)
CMS_AB2	56,566,280	55,530,456 (98.17%)	8.32	94.27	46.78	42,582,890 (76.68%)
CMS_AB3	60,701,770	59,404,212 (97.86%)	8.92	94.09	46.70	44,549,934 (74.99%)

Note: ML_NB and ML_AB represent the normal and abortive bud samples of ML93219, respectively. CMS_NB and CMS_AB represent the normal and abortive bud samples of CMS93219, respectively.

**Table 2:** Summary of de novo transcriptome length distribution.

Length	200–500 bp	500–1 kbp	1 k–2 kbp	>2 kbp	Total	Min (bp)	Mean (bp)	Median (bp)	Max (bp)	N50	Total Nucleotides
No. of transcripts	44,030	21,348	21,467	10,502	97,347	201	936	578	16,361	1510	91,137,323
No. of Unigenes	37,344	12,545	10,691	5470	66,050	201	786	424	16,361	1363	51,896,834

**Table 3:** Annotation of unigene sequences in broccoli buds.

Sequence Database	Number of Annotated Unigenes	Percentage of Annotated Unigene Sequences (%)	Software and Parameters
Annotated in Nr	44,294	67.06	NCBI blast 2.2.28+, *e*-value = 1 × 10^−5^
Annotated in Nt	50,157	75.93	NCBI blast 2.2.28+, *e*-value = 1 × 10^−5^
Annotated in KO	12,403	18.77	KAAS, KEGG Automatic Annotation Server, *e*-value = 1 × 10^−10^
Annotated in SwissProt	29,861	45.2	NCBI blast 2.2.28+, *e*-value = 1 × 10^−5^
Annotated in PFAM	24,660	37.33	HMMER 3.0 package, hmmscan, *e*-value = 0.01
Annotated in GO	30,285	45.85	Blast2GO v2.5 [12] and self-write the script, *e*-value = 1 × 10^−6^
Annotated in KOG	12,492	18.91	NCBI blast 2.2.28+, *e*-value = 1 × 10^−3^
Annotated in all Databases	6070	9.19	-
Annotated in at least one Database	54,753	82.89	-
Total Unigenes	66,050	100	-

**Table 4:** Results of DEGs enriched by Gene Ontology (GO) term.

GO Accession	Description	Term Type	*p* Value	Corrected *p* Value	DEG Item	Gene Names
GO:0071555	cell wall organization	Biological process	9.34 × 10^−10^	3.25 × 10^−6^	9	c23382_g2, c9513_g2, c9513_g1, c24866_g1, c25141_g1, c11939_g1, c54907_g1, c49924_g1, c26161_g1
GO:0045229	external encapsulating structure organization	Biological process	1.34 × 10^−9^	3.25 × 10^−6^	9	c54907_g1, c26161_g1, c49924_g1, c23382_g2, c24866_g1, c25141_g1, c11939_g1, c9513_g1, c9513_g2
GO:0071554	cell wall organization or biogenesis	Biological process	1.66 × 10^−9^	3.25 × 10^−6^	10	c49924_g1, c26161_g1, c52977_g1, c54907_g1, c9513_g1, c9513_g2, c24866_g1, c25141_g1, c11939_g1, c23382_g2
GO:0005985	sucrose metabolic process	Biological process	6.13 × 10^−6^	0.005404	8	c54907_g1, c54637_g1, c23382_g2, c25141_g1, c11939_g1, c24866_g1, c9513_g2, c9513_g1
GO:0005982	starch metabolic process	Biological process	6.46 × 10^−6^	0.005404	8	c23382_g2, c25141_g1, c11939_g1, c24866_g1, c9513_g2, c9513_g1, c54907_g1, c54637_g1
GO:0005984	disaccharide metabolic process	Biological process	8.99 × 10^−6^	0.005849	8	c54907_g1, c54637_g1, c23382_g2, c9513_g2, c9513_g1, c11939_g1, c25141_g1, c24866_g1
GO:0006073	cellular glucan metabolic process	Biological process	1.2 × 10^−5^	0.006395	8	c24866_g1, c11939_g1, c25141_g1, c9513_g2, c9513_g1, c23382_g2, c54637_g1, c54907_g1
GO:0044042	glucan metabolic process	Biological process	1.2 × 10^−5^	0.006395	8	c23382_g2, c9513_g1, c9513_g2, c25141_g1, c11939_g1, c24866_g1, c54907_g1, c54637_g1
GO:0009311	oligosaccharide metabolic process	Biological process	1.66 × 10^−5^	0.008084	8	c23382_g2, c25141_g1, c11939_g1, c24866_g1, c9513_g2, c9513_g1, c54907_g1, c54637_g1
GO:0044264	cellular polysaccharide metabolic process	Biological process	3.33 × 10^−5^	0.013006	9	c54637_g1, c24364_g1, c54907_g1, c24866_g1, c11939_g1, c25141_g1, c9513_g1, c9513_g2, c23382_g2
GO:0005976	polysaccharide metabolic process	Biological process	5.27 × 10^−5^	0.017134	9	c25141_g1, c11939_g1, c24866_g1, c9513_g2, c9513_g1, c23382_g2, c54637_g1, c24364_g1, c54907_g1
GO:0044723	single-organism carbohydrate metabolic process	Biological process	7.34 × 10^−5^	0.022634	11	c11939_g1, c25141_g1, c24866_g1, c9513_g1, c9513_g2, c562_g1, c23382_g2, c17871_g1, c54637_g1, c54907_g1, c24364_g1
GO:0044262	cellular carbohydrate metabolic process	Biological process	0.00015	0.043887	9	c23382_g2, c9513_g1, c9513_g2, c25141_g1, c11939_g1, c24866_g1, c24364_g1, c54907_g1, c54637_g1
GO:0030312	external encapsulating structure	Cellular component	3.87 × 10^−6^	0.005404	6	c23382_g2, c54907_g1, c49924_g1, c11939_g1, c26161_g1, c24866_g1
GO:0005576	extracellular region	Cellular component	7.52 × 10^−6^	0.005507	11	c56244_g1, c28395_g1, c55261_g1, c14539_g1, c49924_g1, c20661_g1, c24866_g1, c25141_g1, c20440_g1, c9513_g2, c9513_g1
GO:0005618	cell wall	Cellular component	2.31 × 10^−5^	0.010397	5	c11939_g1, c26161_g1, c24866_g1, c49924_g1, c54907_g1
GO:0071944	cell periphery	Cellular component	4.68 × 10^−5^	0.016257	8	c49924_g1, c50518_g1, c26161_g1, c54907_g1, c11939_g1, c24866_g1, c23382_g2, c22601_g2
GO:0004650	polygalacturonase activity	Molecular function	6.12 × 10^−6^	0.005404	4	c9513_g1, c9513_g2, c25141_g1, c24866_g1
GO:0004553	hydrolase activity, hydrolyzing O-glycosyl compounds	Molecular function	2.96 × 10^−5^	0.012366	8	c54637_g1, c25141_g1, c24866_g1, c9513_g1, c9513_g2, c23382_g2, c562_g1, c17871_g1
GO:0016798	hydrolase activity, acting on glycosyl bonds	Molecular function	4.72 × 10^−5^	0.016257	8	c17871_g1, c562_g1, c23382_g2, c9513_g1, c9513_g2, c25141_g1, c24866_g1, c54637_g1

**Table 5:** Results of DEGs enriched by Kyoto Encyclopedia of Genes and Genomes (KEGG) pathway.

#Term	ID	Input Number	*p* Value	Corrected *p*-Value	Input
Phenylalanine metabolism	ko00360	3	0.000337	0.006398357	c9433_g1, c20661_g1, c20440_g1
Phenylpropanoid biosynthesis	ko00940	3	0.001398	0.013279387	c9433_g1, c20661_g1, c20440_g1
Propanoate metabolism	ko00640	1	0.042547	0.179678515	c17871_g1
Cutin, suberine and wax biosynthesis	ko00073	1	0.048213	0.179678515	c24364_g1
Fatty acid elongation	ko00062	1	0.051033	0.179678515	c21267_g1
Endocrine and other factor-regulated calcium reabsorption	ko04961	1	0.060379	0.179678515	c22601_g2
alpha-Linolenic acid metabolism	ko00592	1	0.083359	0.179678515	c27465_g3
Photosynthesis	ko00195	1	0.08789	0.179678515	c28693_g1
Synaptic vesicle cycle	ko04721	1	0.090598	0.179678515	c22601_g2
Parkinson’s disease	ko05012	1	0.095095	0.179678515	c25708_g1
Glycerolipid metabolism	ko00561	1	0.104024	0.179678515	c57011_g1
Peroxisome	ko04146	1	0.132037	0.187274571	c24364_g1
Pyruvate metabolism	ko00620	1	0.145727	0.187274571	c17871_g1
Cysteine and methionine metabolism	ko00270	1	0.149117	0.187274571	c17871_g1
Huntington’s disease	ko05016	1	0.161716	0.187274571	c22601_g2
Glycerophospholipid metabolism	ko00564	1	0.164214	0.187274571	c57011_g1
Oxidative phosphorylation	ko00190	1	0.174958	0.187274571	c25708_g1
Glycolysis/Gluconeogenesis	ko00010	1	0.177418	0.187274571	c17871_g1
Endocytosis	ko04144	1	0.208772	0.208772251	c22601_g2

## Supplementary Materials

Supplementary materials can be found at http://www.mdpi.com/1422-0067/19/9/2501/s1. Table S1: Sequences of the specific primers; Table S2: KEGG classification of the unigenes; Table S3: Unigenes expression fold change of CMS_ABvsCMS_NB; Table S4: Unigenes expression fold change of CMS_ABvsML_AB; Table S5: Unigenes expression fold change of CMS_ABvsML_NB; Table S6: FPKM and annotation of the 95 common differentially expressed genes.
